# CD38 inhibitor 78c increases mice lifespan and healthspan in a model of chronological aging

**DOI:** 10.1111/acel.13589

**Published:** 2022-03-08

**Authors:** Thais R. Peclat, Katie L. Thompson, Gina M. Warner, Claudia C.S. Chini, Mariana G. Tarragó, Delaram Z. Mazdeh, Chunlian Zhang, Jose Zavala‐Solorio, Ganesh Kolumam, Yao Liang Wong, Robert L. Cohen, Eduardo N. Chini

**Affiliations:** ^1^ Signal Transduction and Molecular Nutrition Laboratory Kogod Aging Center Department of Anesthesiology and Perioperative Medicine Mayo Clinic College of Medicine Rochester Minnesota USA; ^2^ Department of Anesthesiology and Perioperative Medicine Mayo Clinic Jacksonville Florida USA; ^3^ Calico Life Sciences LLC South San Francisco CA USA

**Keywords:** aging, CD38, healthspan, longevity, mice, NAD, small molecule

## Abstract

Nicotinamide adenine dinucleotide (NAD) levels decline during aging, contributing to physical and metabolic dysfunction. The NADase CD38 plays a key role in age‐related NAD decline. Whether the inhibition of CD38 increases lifespan is not known. Here, we show that the CD38 inhibitor 78c increases lifespan and healthspan of naturally aged mice. In addition to a 10% increase in median survival, 78c improved exercise performance, endurance, and metabolic function in mice. The effects of 78c were different between sexes. Our study is the first to investigate the effect of CD38 inhibition in naturally aged animals.

## Graphical abstract

1. The NADase CD38 plays a key role in age‐related NAD decline.
2. The CD38 inhibitor 78c increases lifespan and healthspan of naturally aged male mice.
3. The increase in median and maximal lifespan was about 10%.

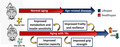

**Abbreviations**
CD38: Cluster of differentiation 3
NAD: Nicotinamide Adenine Dinucleotide

NAD is a cofactor of oxidation–reduction reactions and is a substrate for enzymes involved in cellular homeostasis (Chini et al., 2020; Hogan et al., 2019; Johnson & Imai, 2018; Katsyuba et al., 2020; McReynolds et al., 2020). NAD levels decrease with aging and progeroid states, which is associated with metabolic abnormalities and fitness decline (Camacho‐Pereira et al., 2016; Gomes et al., 2013; Tarrago et al., 2018). The NAD‐consuming enzymes such as CD38 and PARP1 have been shown to play a major role in this process (Aksoy et al., 2006; Camacho‐Pereira et al., 2016; Tarrago et al., 2018). The accumulation of CD38^+^‐inflammatory cells decreases NAD levels in aging (Chini et al., 2019, 2020; Covarrubias et al., 2021). The small molecule 78c is a specific and potent inhibitor of CD38 (Chini et al., 2018; Escande et al., 2013; Tarrago et al., 2018) that boosts NAD levels, improves survival of progeroid mice, and ameliorates several metabolic, structural, and molecular features of aging (Tarrago et al., 2018). However, to date the effect of CD38 inhibition on natural aging and longevity has not been explored. Here, we demonstrate that 78c increases the lifespan and healthspan of naturally aged male mice.

When offered the food to young mice ad libitum, 78c significantly boosted NAD, validating the 78c PO treatment. (Figure S1a). We then placed 1‐year‐old C57BL/6 male and female mice on either a control or 78c diet and closely followed their healthspan and longevity (Figure 1a).

**FIGURE 1:**
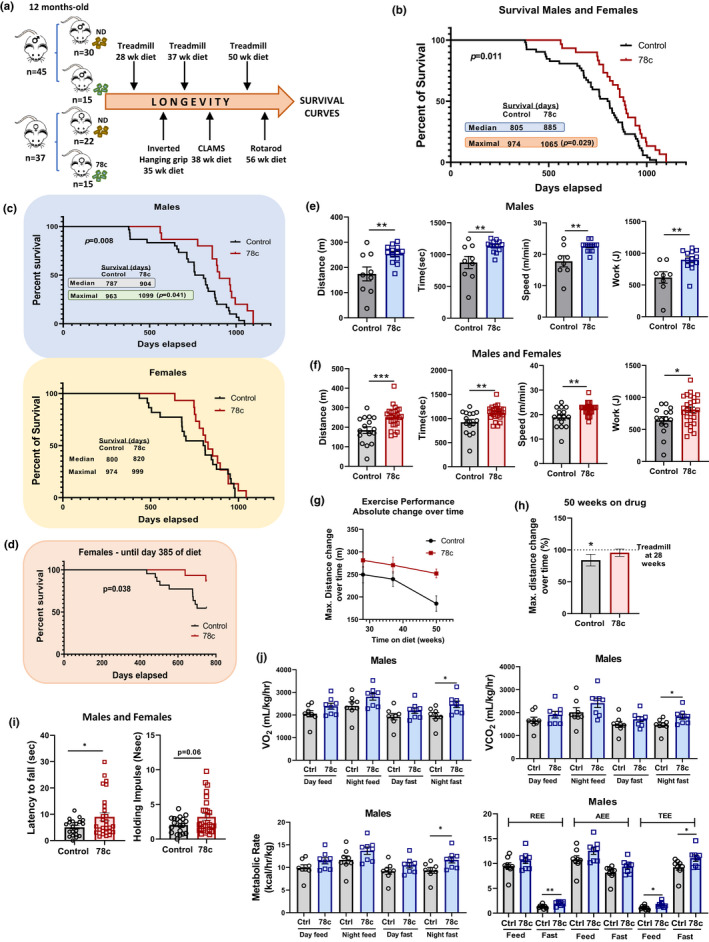
CD38 inhibitor 78c increases lifespan and healthspan in normative aged mice. (a‐j) 1‐year‐old males and females were placed on a control or 78c diet and followed during natural aging (n per group are shown in the scheme). (a) Experiment scheme. (b‐d) Survival curve comparing the control and 78c‐treated mice—(b) all animals, (c) each sex separately, and (d) females only, until day 385 of diet. (e‐f) Uphill treadmill exhaustion test performed at 24 months old (m.o.). Graphs show distance, maximal speed, time, and work. (e) Males only (*n* = 9–14 mice/group), and (f) males and females (*n* = 16–27 mice/group). (g) Maximal distance variation of all animals on different uphill treadmill tests over time. (h) Percent change in maximal treadmill distance at 50 weeks compared to 28 weeks on diet. (i) Inverted four limbs hanging grip test performed at 20 m.o. (*n* = 20–28 mice/group). (j) CLAMS performed on males at 21 m.o. Graphs show VO_2_, VCO_2_, metabolic rate, REE, AEE, and TEE during periods of day and night, and feed and fast (*n* = 8 mice/group). Survival curves were analyzed with log‐rank test. All other data are mean ± SEM and analyzed by unpaired two‐sided t‐test, *p < 0.05, **p < 0.01, ***p < 0.001

When both sexes were grouped, treatment with 78c significantly improved longevity, with a maximal survival increase of 9% (*p* = 0.029) (Figure 1b). When analyzing survival for males and females separately, a sex‐specific effect of 78c was observed. The 78c‐treated males had a 17% increase in median survival (*p* = 0.008) and a 14% increase in maximal lifespan (*p* = 0.041) compared with control. (Figure 1c). In females, no significant survival benefit was observed (Figure 1c).

The causes of death were classified as natural (i.e., animal found dead for unknown reason) and IACUC humane endpoint criteria (Figure S1b). The latter encompasses not only moribund animals but also conditions of non‐fatal health decline that elicit animal suffering. We observed a much higher proportion of IACUC criteria deaths in the 78c‐treated females. Notably, the abrupt decline in the survival curve of that group coincides with the occurrence of consecutive euthanasias due to IACUC recommendation, suggesting that 78c may cause non‐fatal deleterious conditions in female mice. (Figure S1c). Interestingly, the survival analysis up to this point was statistically significant (Figure 1d). In addition, autopsy studies showed no significant difference in the proportion of visible tumors between treated and non‐treated animals (Figure S1d), indicating that 78c increased longevity in males without an evident antitumor effect.

Animals were also longitudinally evaluated for exercise performance, being subjected to a treadmill test at different ages and times on diet (Figure 1a). Males on 78c showed a better exercise performance than their control group starting at 21 months old (m.o.) (37 weeks on diet) (Figure S1e). This difference was maintained in males (Figure 1e) and evident in all animals together (Figure 1f) when they were exercised at 24 m.o. (50 weeks on diet). When evaluating the variation in the maximum distance that animals were able to run to exhaustion over the time, control animals presented a greater negative slope in the curve than the 78c‐treated animals (Figure 1g). After 50 weeks on diet, the control group had significant difference in the percentage of reduction in the achieved maximum distance compared with their performance at 28 weeks of diet and no difference was observed in the 78c‐treated animals (Figure 1h). Muscular strength was also improved, as demonstrated in the hanging grip by a significant higher latency to fall in the 78c‐treated animals compared with control (Figure 1i).

Metabolic parameters were evaluated with Comprehensive Lab Animal Monitoring System (CLAMS) at 21 m.o. Males on 78c presented a significant higher VO_2_, VCO_2_, and metabolic rate during the night fasting. There was a trend to increase in these same parameters during the day fasting and feed times (Figure 1j). Body composition analysis showed that the 78c‐treated animals presented a significant lower percentage of fat and higher lean mass compared to control (Figure 2a), which was not associated with caloric restriction (Figure S1f). Females on 78c showed no significant difference in energy expenditure compared with control (Figure S1g).

**FIGURE 2:**
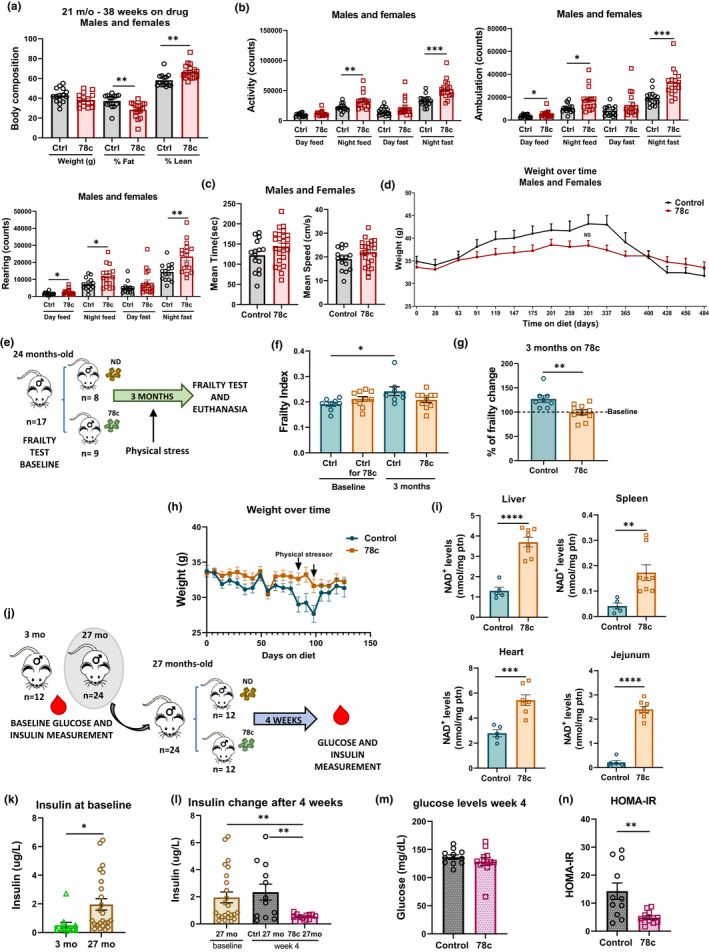
CD38 inhibitor 78c improves healthspan in normative aging. (a‐d) Longevity experiment cohort CLAMS performed on longevity experiment mice. (a) Body composition (weight, % fat, % lean mass) and (b) activity, ambulation, and rearing counts performed at 21 months old (m.o.) (n = 16 mice/group). (c) Rotarod test at 25 m.o. (n = 14–22 mice/group). (d) Weight variation over time. (e‐h) 2‐year‐old male mice were placed on a control or 78c diet and followed for 3 months. (e) Experiment scheme. (f) Frailty index and (g) % change in frailty index after 3 months (n = 8–10 mice/group). (h) Weight variability over time (n = 9–13 mice/group). (i) NAD levels in tissues. (j‐n) 3 and 27 m.o. males underwent glucose and insulin measurement. Then, 27 m.o. mice were placed on a control or 78c diet for 4 weeks (n = 12 mice/group). (k) Baseline insulin levels. (l‐n) insulin, glucose, and HOMA‐IR from 27 m.o. mice after 4 weeks on a control or 78c diet. Data are mean ± SEM, analyzed by unpaired two‐sided t‐test, *p < 0.05, **p < 0.01, ***p < 0.001, ****p < 0.0001

Especially during the night, 78c treatment significantly increased activity, ambulation, and rearing counts (Figure 2b), but rotarod performance showed no statistically significant differences (Figure 2c). The control group presented an abrupt decline in their weight with aging, which is one indication of frailty. By contrast, the 78c‐treated animals had a steadier variation in the weight curve throughout the whole experiment (Figure 2d).

We then evaluated the effect of 78c on the frailty in a cohort of old male mice (Figure 2e). Frailty scores were derived from clinical examination (Whitehead et al., 2014). Changes in frailty index after 3 months were plotted in comparison with the baseline index (Figure 2f). All animals in the control group had a significantly higher frailty index than that was 3 months earlier, which occurred mainly due to a worsening in grimace, body condition score, kyphosis, tremor, and eye discharge. By contrast, 78c showed a protection against age‐related frailty increase (Figure 2g). Physical exhaustion on treadmill was used as an additional stressor to determine resilience. The weight curve shows a clear pattern of weight preservation and better weight recovery after the two timepoints of physical stress in the animals treated with 78c compared with control (Figure 2h). 78c promoted increase in NAD levels (Figure 2i) but it did not change the expression of senescence markers (Figure S1h), suggesting that NAD boosting started at a later age ameliorates aging through mechanisms other than decreasing senescence. Finally, we observed that the treatment of old male mice with 78c for 4 weeks improved insulin levels and sensitivity (HOMA‐IR) to the levels similar to young mice (Figure 2j‐n) and that this effect was independent of body weight and food intake (Figure S1i‐j).

Our results represent the first *in vivo* longitudinal study using a CD38 inhibitor in natural aging. Oral administration of 78c allows a steady dose delivery, avoiding potential complications related to intraperitoneal injections. Therefore, this approach improves the translational potential of CD38 inhibitors as a therapy for age‐related diseases, promoting healthier aging.

## CONFLICTS OF INTEREST

E.N.C. holds a patent on CD38 inhibitors licensed by Elysium health. E.N.C. consults for Calico, Mitobridge, and Cytokinetics. Others declare no conflicts.

## AUTHOR CONTRIBUTIONS

TRP, ENC, RLC, GK, and YLW conceived the study. TRP, ENC, GMW, and CCSC designed the study. TRP, KLT, DZM, MGT, CZ, and JZS carried out the experiments. All authors drafted the manuscript.

## Supporting information

Fig S1

Supplementary Material

Supplementary Material

## Data Availability

The data that support this study are available from the corresponding author upon reasonable request.
