# Supplementary figures and images for: CD38 inhibitor 78c increases mice lifespan and healthspan in a model of chronological aging
Source: Aging Cell. 2022 Mar 8;21(4):e13589. doi: 10.1111/acel.13589 (PMC9009115; doi:10.1111/acel.13589)

## Slide 1
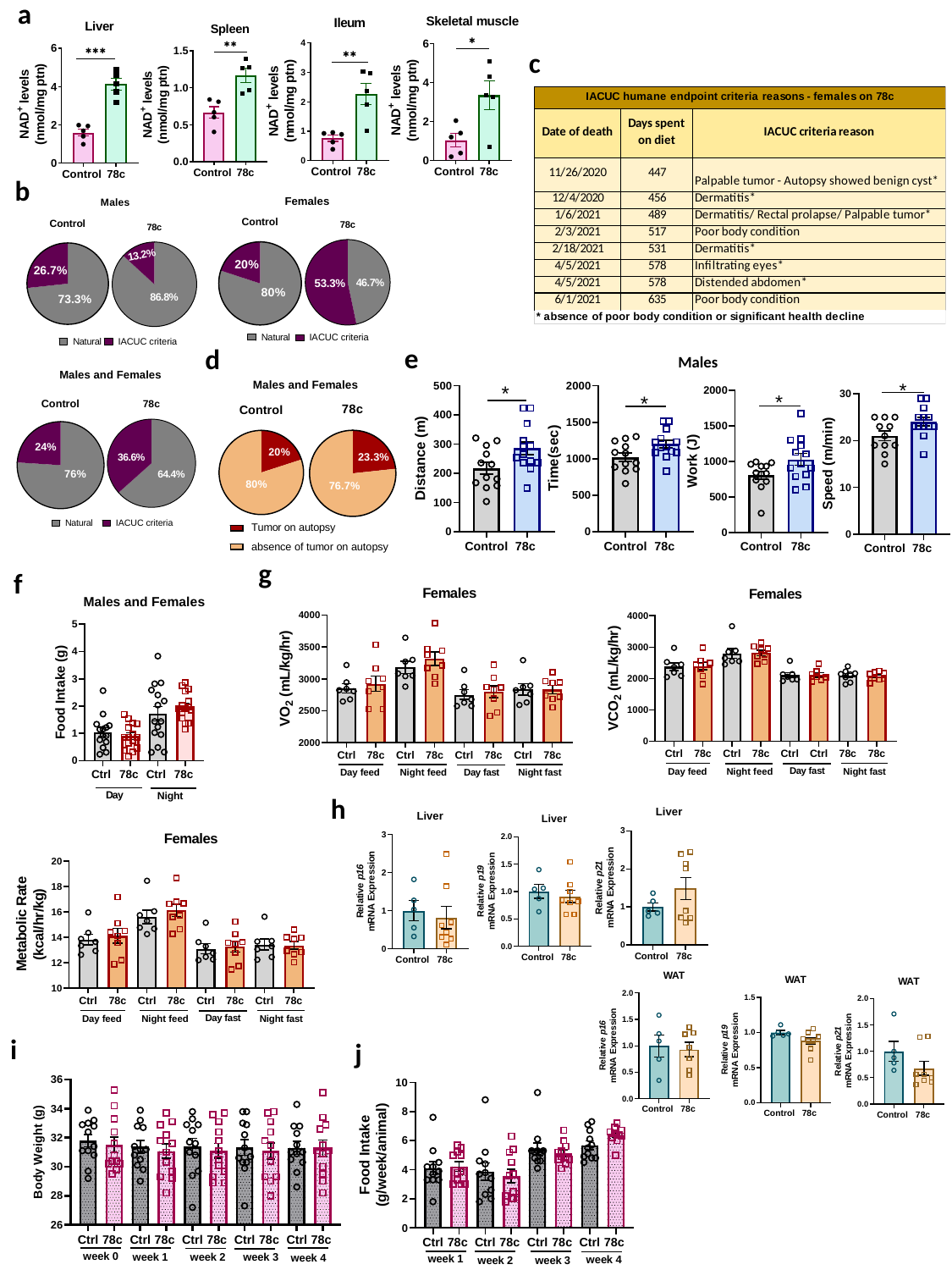

a
c
b
e
d
Males
g
f
h
i
j

Supplement: Supplementary file 1 — Fig S1 [file ACEL-21-e13589-s003.pptx]
