# Supplementary material for: CD38 inhibitor 78c increases mice lifespan and healthspan in a model of chronological aging
Source: Aging Cell. 2022 Mar 8;21(4):e13589. doi: 10.1111/acel.13589 (PMC9009115; doi:10.1111/acel.13589)
Supplement: Supplementary file 2 — Supplementary Material [file ACEL-21-e13589-s001.docx]

**Supplementary information:**

**MATERIALS AND METHODS**

1. **Animals**

All protocols requiring animal manipulation in the USA were approved by the Mayo Clinic Institutional Animal Care and Use Committee (IACUC), the Calico Lifescience Institutional Animal Care and Use Committee (IACUC), and the studies were carried out in adherence to the NIH Guide for the Care and Use of Laboratory Animals (IACUC protocol #: A00003888). Male and female mice were used for these experiments. They were housed in standard cages at room temperature and constant humidity, with a 12-hour light-dark cycle, and maintained on a normal chow diet (ND) (Envigo global 14% protein diet TD.00357) or a normal diet enriched with 78c (Envigo 2014+600ppm x307610 TD.180858) ad libitum. Three different cohorts were used to complete the experiment. Figures 1a, 2e and 2i show a schematic representation of the experiment design for each cohort. Animals were weighted once a week and food intake was also followed weekly for the total duration of each experiment.

1. **Healthspan evaluation Tests**
   1. **Treadmill exhaustion test**

Exercise capacity was assessed by measuring running time, distance, maximal running speed, and work using a motorized treadmill (Columbus Instruments) in the different experiments performed as described^1,2^. The mice were acclimated to the treadmill for 3 consecutive days, 5 minutes per day, at a speed of 10 m/min and a grade of 5%. After a day of rest, animals ran on the treadmill at an initial speed of 5 m/min and 5% grade for 2 minutes, after which the speed was increased by 2 m/min every subsequent 2 minutes until the mice were exhausted. Exhaustion was defined as the inability of the mouse to remain on the treadmill despite an electrical shock stimulus.

Running time was recorded, running distance was calculated by the software (Columbus Instruments), and work (the product of body weight [kg], gravity [9.81 m/s2], vertical speed [m/s × angle], and time [s]) was calculated^1,2^ .

- 1. **Four limbs Inverted Hanging grip**

The four limbs hang test uses a wire grid system to non-invasively measure the ability of mice to exhibit sustained limb tension to oppose their gravitational force. The procedure measures the 4 limbs hang time in seconds as well as the minimal Holding impulse (Holding Impulse=Body mass x Hang Time) that is used to oppose the gravitational force. It is an efficient and reliable outcome measure for the evaluation of the effects of potential therapeutic compounds on muscle strength. Mice are clearly willing to perform the test since behavior indicates that they do not want to fall off the grid. The Holding Impulse is used as an attempt to correct for the negative effects of body mass on the Hang Time. The four limbs hang test can be performed using the top of a cage (tops of rat cages work well). Soft bedding must be placed underneath the grid to break the fall and prevent the mice from harming themselves. There is no need for training before the test day, however, mice are acclimated to the test room one hour before starting the test.^3^

- 1. **Rotarod**

It is a performance test based on a rotating rod with forced motor activity being applied. The test measures parameters such as riding time (seconds) or endurance. In the test, a rodent is placed on a horizontally oriented, rotating cylinder (rod) suspended above a cage floor, which is low enough not to injure the animal, but high enough to induce avoidance of fall. Rodents naturally try to stay on the rotating cylinder, or rotarod and avoid falling to the ground. The length of time that a given animal stays on this rotating rod is a measure of their balance, grip strength, coordination, physical condition, and motor-planning. The speed of the rotarod is mechanically driven. Mice are trained during 3 consecutive days for 5 minutes of a constant acceleration of 4 mm/s (day 1), 6 mm/s (day 2), and 8 mm/s (day3). The fourth day is test day and mice are tested in a ramp mode, with an initial acceleration of 4 mm/s and final acceleration of 40 mm/s with a maximal of 5 minutes of the test. In the test, day mice are tested 3 times. On all days, mice are acclimated to the Rotarod room one hour before starting.

- 1. **Frailty**

Frailty is based on a 31-items performance-based frailty index that reflect on clinical signs of deterioration in mice^4^. These clinical assessments include placing one mouse at a time on wrist to analyze: eyes swelling/secretions, color and care of the fur, tail stiffening (by sliding a finger through the ventral side of the tail). Mouse is then hanged by its tail and transfer to a transparent cage to analyze the vestibulocochlear system (by hanging mice by the tail, we see if they rotate their heads) and grasping score (neurological assessment of grasping limbs ventrally) - It takes less than 2 minutes hanging by the tail. We slowly approach the mouse to the cage and analyze their paws reaction to reach the cage floor, before whiskers touch the cage, as a vision evaluation. We let the mouse ambulate through the cage and analyze any gaiting disorder, while mice calm down from handling. After around 2 minutes we analyze grimace scale, piloerection and breathing abnormalities. Then we test the auditory system (using a clicker and observing reactions at ears, whiskers and/or freezing movement), weight the mouse using a balance, and pick the mouse by its back. We observe nasal swelling/secretion, abdomen distention, abnormalities at the urogenital system, and touch the abdomen for signs of tumors. Using a laser thermometer, we measure the body surface temperature aiming at the abdomen 3 times (°C).

The severity of each parameter was rated with a simple scale: a score of 0 will be given for mice displaying no sign. For mild deficits, a score of 0.5 was given and for severe sign, a score of 1 was recorded in a printed paper checklist. The test was conducted together with a Grip Strength. Frailty index is calculated dividing the total frailty score of each animal by 31 (number os parameters evaluated).

**2.5 Comprehensive Laboratory Animal Monitoring System**

Metabolic rates are measured at room temperature by indirect calorimetry of single animals in open-circuit ‘‘oxy-max’’ Comprehensive Laboratory Animal Monitoring System (CLAMS) chambers (Columbus Instruments)^5^. On the day of the experiment, mice are weighed and acclimated in the chambers overnight. Over the next 48 hours, locomotor activity, oxygen consumption (VO2), and CO2 production (VCO2) are monitored in real time. Mice have food and water ad libitum during the first 24 hours and are fasted during the subsequent 24 hours.

1. **Glucose, insulin, and HOMA-IR**

For glucose and insulin measurements, animals were fasted for 3 hours in the morning. Blood was collected through retro-orbital route. Glucose was measured using handheld glucose meter (Onetouch). Serum was used for Insulin assay using ultra-sensitive mouse insulin ELISA kit (Mercodia, cat no: 10-1247-01). HOMA-IR was calculated was calculated using the equation [(G_0_ x I_0_)/405)], where G_0_ and I_0_ refer to fasting plasma glucose and insulin, respectively.

1. **Post-mortem evaluation**

Mice were removed from the study either because found dead in their cage or when assigned to be euthanized due to IACUC criteria. A brief necropsy was done for all animals that were found in adequate conditions, i.e. animals that were either euthanized for IACUC criteria or found dead in their cages not much longer after their death. Some necropsies were impaired due to post-mortem cannibalism. Necropsies were focused on screening for visible tumors in the abdomen, thoracic cavity, brain, limbs, and soft tissue. All found solid masses were counted as tumors. Tissues were not sent for histological analysis by a pathologist.

1. **Measurement of NAD+ levels by Cycling Assay**

Detection of NAD^+^ was performed as described before using a cycling assay (). Between 15 to 20 mg of tissue was homogenized in 10% trichloroacetic acid (TCA). Samples were centrifuged at 12,000 rpm for 2 min at 4°C. The supernatants were collected, and the pellets were resuspended in 0.2 M NaOH for protein quantification. TCA was removed with organic solvents (3 volumes 1,1,2-trichloro-1,2,2-trifluroethane: 1 volume trioctylamine) in a ratio of 2 volumes of organic solvent to 1 volume of sample. The resulting top aqueous layer containing NAD+ was recovered and 1M Tris pH 8.0 was added for the pH correction. Samples were then diluted in 100 mM sodium phosphate buffer pH 8 in a volume of 100 μL/ well and added to white 96 well plates. For the cycling assay, 100 μL of reaction mix (0.76% ethanol, 4 μM FMN, 27.2 U/ mL alcohol dehydrogenase (ADH), 1.8 U/ mL Diaphorase, and 8 μM resazurin) was added to each well. 96-well plates were read in a fluorescence plate reader (Molecular Devices, SpectraMax® Gemini™ 1579 XPS) in an excitation wavelength of 544 nm and an emission wavelength of 590 nm^6,7^.

1. **Statistical Analysis**

Data were expressed as the mean ± SEM. Statistical analyses were performed using unpaired, two-tailed Student’s t test for two populations’ means at a single time point. Survival was analyzed according to the Kaplan-Meier method, and differences in their distribution were evaluated by the log-rank (Mantel-Cox) test and the Gehan-Breslow-Wilcoxon test. Maximal survival was calculated as the mean of the 90^th^ quantile for each cohort. Quantile regression modeling was used to calculate the difference between groups for maximal lifespan. p values < 0.05 were regarded as significant. Analyses were performed using GraphPad Prism 9. IBM SPSS Statistics version 28 was used for statistical analysis of the maximal lifespan.

**References**

1 LeBrasseur, N. K. *et al.* Myostatin inhibition enhances the effects of exercise on performance and metabolic outcomes in aged mice. *J Gerontol A Biol Sci Med Sci* **64**, 940-948, doi:10.1093/gerona/glp068 (2009).

2 Tarrago, M. G. *et al.* A Potent and Specific CD38 Inhibitor Ameliorates Age-Related Metabolic Dysfunction by Reversing Tissue NAD(+) Decline. *Cell Metab* **27**, 1081-1095.e1010, doi:10.1016/j.cmet.2018.03.016 (2018).

3 Deacon, R. M. Measuring the strength of mice. *J Vis Exp*, doi:10.3791/2610 (2013).

4 Whitehead, J. C. *et al.* A clinical frailty index in aging mice: comparisons with frailty index data in humans. *J Gerontol A Biol Sci Med Sci* **69**, 621-632, doi:10.1093/gerona/glt136 (2014).

5 Izumiya, Y. *et al.* Fast/Glycolytic muscle fiber growth reduces fat mass and improves metabolic parameters in obese mice. *Cell Metab* **7**, 159-172, doi:10.1016/j.cmet.2007.11.003 (2008).

6 Camacho-Pereira, J. *et al.* CD38 Dictates Age-Related NAD Decline and Mitochondrial Dysfunction through an SIRT3-Dependent Mechanism. *Cell Metab* **23**, 1127-1139, doi:10.1016/j.cmet.2016.05.006 (2016).

7 Aksoy, P. *et al.* Regulation of SIRT 1 mediated NAD dependent deacetylation: a novel role for the multifunctional enzyme CD38. *Biochem Biophys Res Commun* **349**, 353-359, doi:10.1016/j.bbrc.2006.08.066 (2006).
