# Supplementary material for: CD38 inhibitor 78c increases mice lifespan and healthspan in a model of chronological aging
Source: Aging Cell. 2022 Mar 8;21(4):e13589. doi: 10.1111/acel.13589 (PMC9009115; doi:10.1111/acel.13589)
Supplement: Supplementary file 3 — Supplementary Material [file ACEL-21-e13589-s002.docx]

**Supplementary Figure 1:**

(a) 3-month old (m.o.) animals were placed on a control or 78c diet for 1 week (n=5 mice/group). NAD levels were measured in liver, spleen, ileum and skeletal muscle. (b-g) 1 year-old animals (males and females) were placed on a control or 78c diet and followed during natural aging. (b) Pie charts showing the distribution of natural and IACUC humane endpoint criteria deaths in all animals together and by sex. (c) Table showing the specific reasons for IACUC humane endpoint criteria in the female group treated with 78c. (d) Pie chart showing the percentage of tumors found in the animals’ autopsies in each group. (e) Uphill treadmill exhaustion test performed on males after 37 weeks on diet when animals were 21 m.o. (n=12-13 mice/group). Graphs show distance, maximal speed, time, and work until exhaustion. (f) Food intake of all animals during the Comprehensive Lab Animal Monitoring System (CLAMS) done when at 39 weeks on diet and 21 m.o. (n=8 mice/group). (g) CLAMS on females (39 weeks on diet and 21 m.o.). Graphs show VO_2_, VCO_2_, and Metabolic rate (n=8 mice/group). (h) Relative mRNA expression of *p16*, *p19* and *p21* measured by qPCR in samples from 2-year-old male mice placed on a control or 78c diet for 3 months. (i-j) 27 m.o. males were placed on a control or 78c diet and followed for 4 weeks (n=12 mice/group). (i) Body weight. (j) Food intake. Data are mean ± SEM, analyzed by unpaired two-sided t-test, *P<0.05, **P<0.01, ***P<0.001.
